# Microbiological Quality of Milk Donated to the Regional Human Milk Bank in Warsaw in the First Four Years of Activity

**DOI:** 10.3390/healthcare10030444

**Published:** 2022-02-26

**Authors:** Kamila Strom, Sylwia Jarzynka, Anna Minkiewicz-Zochniak, Olga Barbarska, Gabriela Olędzka, Aleksandra Wesolowska

**Affiliations:** 1 Department of Medical Biology, Faculty of Health Sciences, Medical University of Warsaw, 14/16 Litewska St., 00-575 Warsaw, Poland; kamila.strom@wum.edu.pl (K.S.); sylwia.jarzynka@wum.edu.pl (S.J.); anna.minkiewicz@wum.edu.pl (A.M.-Z.); o.barbarska@gmail.com (O.B.); gabriela.oledzka@wum.edu.pl (G.O.); 2 Laboratory of Human Milk and Lactation Research at Regional Human Milk Bank in Holy Family Hospital, Department of Medical Biology, Faculty of Health Sciences, Medical University of Warsaw, 14/16 Litewska St., 00-575 Warsaw, Poland

**Keywords:** regional human milk banks, human milk, microbiological safety, donor human milk

## Abstract

As the survival rate for preterm infants increases, more emphasis is placed on improving health-related quality of life through optimal nutritional management. Human Milk Banks (HMBs) provide bioactive nutrients and probiotic microorganisms to premature newborns, especially in the first year of life. Donated milk screening and selection of potential donors ensures the quality and microbiological safety of the donated milk. Therefore we reviewed the basic characteristics of donors and the amounts and contamination of breast milk donated to the Regional Human Milk Bank (RHMB) in Warsaw. In four years, the RHMB collected 1445.59 L of milk, of which 96.60% was distributed among hospitalised infants. Additionally, breastmilk from donor candidates (139 samples from 96 women) was tested at least once in the first year of lactation. First analyses showed that 18 women’s milk samples were microbiologically pure, and 78 samples had one or more species of commensal and/or potentially pathogenic bacteria. In human milk samples from 10 women, the bacteria level was above the standard required by the RHMB; therefore, donors were re-educated, and further samples were tested. Most women followed the recommendations on hygienic expression and storage of milk before transfer to the RHMB. Our analysis will help to increase the accessibility and quality of raw donor milk and to meet the needs of more newborns.

## 1. Introduction

Human Milk (HM) is a unique composition of nutrients, ensuring proper development and shaping the intestinal microbiome of infants. It contains all necessary nutritional and bioactive components, such as growth factors, immunological factors and oligosaccharides [1]. This complex biofluid modulates the immune system of the newborn, reducing the risk of diseases, including necrotizing enterocolitis, respiratory tract infections, atopy, or late-onset sepsis [2,3,4]. It is recommended by the World Health Organization (WHO) for exclusive feeding of newborns up to 6 months of age, and later, together with complimentary food up to 2 years of age or longer. In the absence of a mother’s own milk, extremely preterm infants should be fed donor human milk (DHM) [4]. According to current knowledge, HM is not a sterile liquid. It is a reservoir of microorganisms: pathogenic, commensal, and probiotic. In addition, research indicates the presence of fungi and viruses in breast milk [5,6]. Around 700 strains of bacteria have been isolated from human milk so far. The most frequently isolated microorganisms from milk belonged to phyla *Proteobacteria* and *Firmicutes*; genera *Staphylococcus*, *Streptococcus*, *Propionibacterium* [3,7,8]. In addition, probiotic *Lactobacillus* and *Enterococcus*, derived from skin and *Bifidobacterium,* and, less frequently, *Enterobacteriaceae*, *Rothia, Corynebacterium,* and *Leuconostoc* have been isolated from HM samples. In contrast, some potential pathogenic bacteria in human milk are *Escherichia coli*, *Staphylococcus aureus*, Group B *Streptococci*, *Klebsiella pneumoniae*, *Enterobacter sakazakii*, (β-hemolytic) *Streptococcus pyogenes*, and species of *Pseudomonas*, *Proteus,* and *Salmonella* sp. [9,10,11,12]. Despite the fact that these bacteria are potentially pathogenic, they are the natural microbiome of human milk, because they are isolated in the fresh milk of healthy women from all over the world [13].

Regional Human Milk Banks (RHMBs) are institutions that have been operating in Poland since 2012. Currently, there are 16 such institutions in large Polish cities, to which honorary human milk donors donate surplus milk [14]. the European Milk Bank Association (EMBA) gives some recommendations for principles of operation of such facilities in Europe [15]. However, every European country has significantly divergent practices regarding optimal testing regimes, either before or after pasteurization, and detailed, consistent HMB guidelines are lacking even within countries. In Poland, every RHMB is affiliated with the Human Milk Bank Foundation, which, in association with experts appointed by the Chief Sanitary Inspector, has published guidelines in order to regulate operations of RHMB [14,15,16,17].

A donor candidate must be a healthy woman who effectively breastfeeds her offspring and is willing to donate surplus milk free of charge. Women who successfully pass the epidemiological recruitment receive instructions on the principles of aseptic milk expression, the rules of disinfection and cleaning of the lactation equipment, and storing the milk until it is delivered to the appropriate facility. In the Regional Human Milk Bank in Warsaw, milk from donors is subject to detailed examination during recruitment. Those practices ensure the quality and microbiological safety of the donated milk. Although the milk from donors is thermally pasteurized, microbiological analysis of the first samples is necessary because testing for microbial contamination makes it possible to discard all milk samples that do not meet standards according to the recommendations. After each donor is successfully recruited, she is scheduled to perform a control analysis every 3 months. If a donor fails the initial recruitment, her qualification is determined by a positive result from the newly taken sample. Donated milk after holder pasteurization (62.5 °C, 30 min) must be microbiologically clean; then, milk is kept in the freezer until distribution (up to 3 months) [14,15].

The women, for whom none of the samples meet the requirements, must be rejected. Therefore, it is important to assess the number of these samples, detect possible factors/reasons for overly high levels of bacteria, and implement procedures that will prevent wasting milk or unnecessary rejection of potential donors. The aim of this study was to summarize the microbiological data collected by the Regional Human Milk Bank in Warsaw in 2016–2019 regarding the information on candidates for donors and the microbiological purity of milk donated by them.

## 2. Materials and Methods

Statistical data were collected by the Regional Human Milk Bank (RHMB) in Warsaw in 2016–2019 regarding the microbiological analyses of milk from new 96 donors. Microbiological testing was performed by an external laboratory, Diagnostyka S.A. in Warsaw, in accordance with the routine microbiological procedures in force in the unit, towards establishing the microbiota and potentially pathogenic microorganisms, including aerobic and anaerobic bacteria and/or fungi. Each sample of milk was inoculated with the calibrated loop on Columbia Agar, UTI Chromogenic Agar, Mannitol Salt Agar, Saburaud Agar, and *Bacillus cereus* Selective Medium (PEMBA). All plates were placed in an incubator for 48 h at 37 °C. The bacterial growth was analysed after the incubation time, including colony count, the morphology of colony, and types of hemolysis. Microorganism identification to the species level was performed by MALDI Biotyper Bruker (Bruker Daltonik GmbH, Germany) and VITEK^®^ 2 Compact (bioMérieux, France). In addition, during identification, additional tests were also used, e.g., agglutination test, oxidase test, catalase test, and Gram stain.

The Regional Human Milk Bank, according to the internal recommendation, set the total number of bacteria in the sample as <10^5^ CFU/mL as the basic milk standard, the number of coagulase-positive staphylococci as <10^4^ CFU/mL, and the number of coliform bacteria as <10^3^ CFU/mL [14]. The research was carried out on 139 samples of human milk donated by 96 candidates for human milk donation. Each woman had at least one milk analysis done. In case the level of the analysed microorganisms was above the threshold adopted by the bank or there were doubts concerning the patient’s health (e.g., mastitis), the donor had a new analysis done. At least one week after the first examination, tests were repeated on the newly taken samples. Some donors were scheduled to perform a control analysis after 3 months. Repeated analyses were performed for 31 women (43 samples).

All data were statistically analysed, and the analyses were performed using Microsoft Excel 365 (Washington, DC, USA) licensed to the Medical University of Warsaw.

## 3. Results

### 3.1. Characteristics of Candidates

**Figure 1:**
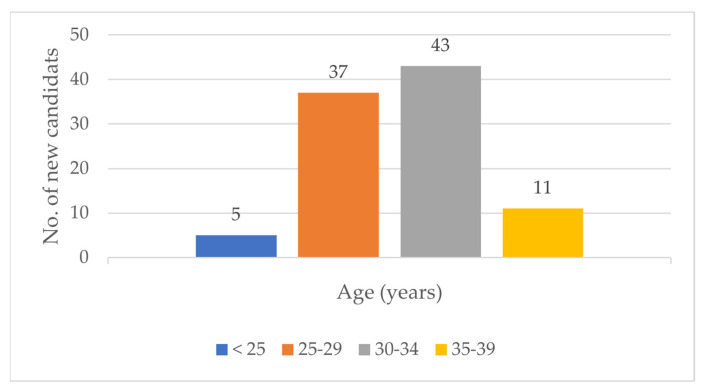
Age groups of candidates for human milk donation.

In the years 2016–2019, 96 new candidates for human milk donation were registered in the RHMB. The average age of women who reported to the Regional Human Milk Bank was 30.8 years. The youngest was 20 years old and the oldest was 39. Detailed data are included in Figure 1.

In 2016 twenty-five mothers applied to RHMB, in 2017–twenty, in 2018–twenty-six, and in 2019–twenty-five. On average, donors came 32 days after the birth of their child. 78.13% came in the first three months of lactation, 16.16% between 4th and 6th, 2.08% between 7th and 9th, and 1.04% 10–12 months of lactation. 2.08% of women reported in the 12th month of lactation and later. 

In total, 1445.59 L of milk were collected during four years of operation of the Regional Human Milk Bank. 96.60% of the milk collected by the RHMB was distributed among hospitalized premature infants. 31.11 L of milk from donors were disposed of due to the failure of the pasteurizer. All data are in Table 1.

### 3.2. Microbiological Evaluation of Milk Samples

**Figure 2:**
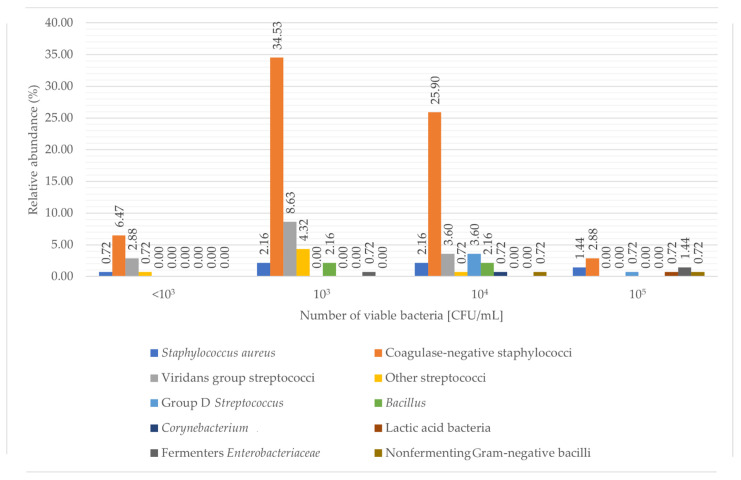
Quantitative results of detected groups of microorganisms in 139 milk samples. The data are presented as a percentage of samples with a certain number of viable bacteria. We found 93.52% of samples without growth of *Staphylococcus aureus*, 30.22% without coagulase-negative staphylococci, 84.89% without viridans group streptococci, 94.24% without other streptococci, 95.68% without group D *Streptococcus*, 95.68% without *Bacillus*, 99.28% without *Corynebacterium*, 99.28% without lactic acid bacteria, 97.84% without fermenters *Enterobacteriaceae*, and 98.56% without nonfermenting Gram-negative bacilli (data not shown in figure).

In total, 139 milk samples were microbiologically tested. Among the samples collected by the Regional Human Milk Bank in Warsaw, the amount of detected microorganisms ranged from 10^0^ to 10^5^ CFU/mL (Figure 2). The median bacterial count was 10^3^ CFU/mL. During the preliminary study, 18 samples from the studied group of 96 women were microbiologically clean, 43 milk samples contained one species of bacteria, and 35 samples had more than one species of commensal and/or pathogenic bacteria. Re-analyses of newly collected samples were performed for 31 women. In 10 of them, the microbiological results during the initial analysis exceeded the quantitative standard adopted by the RHMB, because the results of the milk tests and/or health status raised other concerns (e.g., diagnosed mastitis on the day of feeding, infection). 21 donors had standard control analysis after 3 months.

Repeated testing of newly collected milk samples showed that most of the women followed the advice on how to express and store their milk properly. This is confirmed by the quantitative data for each group of bacteria (Table 2). Among the re-examined group, 36 samples were accepted, while in 7 samples the norm was exceeded for at least one species of bacteria. In repeated studies, the greatest reduction in bacteria was found among staphylococci. Although *Staphylococcus epidermidis* was diagnosed in 25 samples, none of them had a disqualifying level. The remaining groups of microorganisms were grown in excess of the norm only in single samples.

During microbiological analysis, 24 different bacteria were identified (data presented in Table 3). The most frequently detected microorganisms in the preliminary study were *Staphylococcus epidermidis*, which was detected in 72.92% of the tested women, *Streptococcus mitis/oralis,* in 12.50%, and *Staphylococcus aureus,* in 7.29%. These data coincided with the statistics from repeated studies, where the most frequently detected bacteria were those from the coagulase-negative staphylococci group. The most frequently detected microorganisms were *Staphylococcus epidermidis* (58.14%). The remaining data are presented in Table 3. Additionally, each milk sample was tested for the presence of fungi, and only *Candida albicans* were detected. Growth on the medium was found only in one patient, in which single colonies had grown.

## 4. Discussion

The development of Regional Human Milk Banks in Poland and the rest of the world has happening continuously for many years. They give willing volunteers the ability to donate surplus milk [15,16,18]. The amount of milk donated to banks depends primarily on the number of active donors. In our statistics, we provided candidates for human milk donation without comparing values from other institutions. However, in most banks around the world, it can be seen that the amount of donated milk is increasing every year, which proves the increase in the popularity of milk donation in official establishments and the increasing awareness of the value of breast milk [19,20,21,22,23].

Regional Human Milk Banks place a great deal of importance on the quality of the milk they distribute to premature infants. An important factor that is taken into account when qualifying milk to the bank is its microbiological purity. Despite the fact that EMBA’s recommendation was published in 2019 [15], in our RHMB, similar microbiological norms were established earlier. EMBA recommends that donor human milk (DHM before pasteurization should contain ≤ 10^5^ CFU/mL non-pathogenic organisms and no pathogens for each DHM sample during the recruitment of donors [15]. In our RHMB, the total number of bacteria in the sample was <10^5^ CFU/mL, the number of coagulase-positive staphylococci was <10^4^ CFU/mL, and the number of coliform bacteria was <10^3^ CFU/mL [14]. The standards adopted by the RHMB were exceeded by 31 women in the first stage but only by 7 women in repeated studies. The applicants’ failure to pump hygienically could be a major reason why their milk was not approved, because the majority of bacteria come from the microbiome of the mother’s skin. On the other hand, such a high final recruitment result confirms that the recommendations and instructions provided at the recruitment stage bring beneficial effects and that the milk provided by the donor is of high quality.

It is recommended that each European country standardizes its own guidelines for assessing the contamination of donor milk according to EMBA rules [15]. In Poland, every single RHMB follows internal procedures, but unfortunately, they are not monitored on a national level. National guidelines, as well as a central governing body for HMBs, would be beneficial [17]. In Poland, there are many non-government organizations that promote breastfeeding. The Human Milk Bank Foundation is the only official representative of the EMBA, and therefore it evaluates the standards of RHMBs operating in Poland. This institution assumes patronage over RHMBs and promotes safe donating of HM, regardless of the place of residence and the economic status of the family of preterm infants [14]. Across Europe, huge variation in the microbiological acceptance criteria and the microbiological screening practices of unpasteurised and pasteurised DHM exist. The most common method is culture testing [17]. A good practice at the RHMB in Warsaw is to analyse the grown colonies on selective and differential media, with automatic methods identifying microorganisms with high accuracy. Evaluation by MALDI Biotyper Bruker (Bruker Daltonik GmbH, Germany) can be performed directly from a single colony grown on culture plates. This novel method is an excellent method for microorganism identification and gives results quickly, which is important during the recruitment of new donors. Schulthess et al. [24] showed that using the MALDI Biotyper is highly reliable, keeping in mind that identification rates can be lower in the case of some Gram-positive or Gram-negative cocci. For this reason, in our RHMB, we performed additional biochemical tests for bacteria.

In order to exclude the risk of administering contaminated milk to a newborn, the HMB uses the holder pasteurisation for milk preservation. This technique guarantees the microbiological safety of milk [25]. The EMBA recommends analyses of all pools of milk before pasteurization, and of each bath after holder pasteurization [15]. In the RHMB in Warsaw, these rules are followed. Additionally, a good approach of the Polish RHMB is that the tested samples from only a single donor are pooled, in contrast to some countries where multi-donor pooled milk is analysed. This practice eliminates the problem of disposing of large volumes of pooled milk. In addition, great emphasis is placed on long-term cooperation with nursing women, thanks to which the milk they donate is less contaminated because they are better educated.

Human milk, in addition to health-promoting bacteria, contains these potential pathogens [26]. Possible exposure to pathogenic bacteria may contribute to the development of newborn sepsis, meningitis, and necrotizing enterocolitis, and diarrhoea, especially for premature babies and immunocompromised infants [10]. We found some pathogens in our samples, like *S. aureus, B. cereus, E. coli* (respectively, 6.47%, 4.32%, and 0.72% of all analysed samples). In the samples collected by the RHMB in Warsaw major pathogens were not isolated according to the EMBA, such as *Cronobacter sakazakii* and *Listeria monocytogenes*.

The pathways by which microorganisms enter milk are different. The traditional theory suggests that microorganisms enter human milk not only from the mother’s skin, inhabited by the physiological microbiome, but also due to environmental contamination [6]. The most numerous species were *Bacillus*, *Acinetobacter*, *Enterobacteriaceae*, *Pseudomonas*, *Staphylococcus*, and *Propionibacterium* [27]. *Streptococcus* bacteria enter the milk possibly by the retrograde flow of milk from the infant’s mouth back to the female mammary gland. An increased amount of *Staphylococcus aureus* in the sample could be associated with mastitis [11,12]. Pumping equipment may be another source of contamination of donated milk. Jimenez et al. [28] suggested such a theory, based on the observation that women who pumped manually had fewer bacteria than those using the appropriate devices. This was most evident in *Enterobacteriaceae*, other Gram-negative bacteria, and *Candida*. Their levels were significantly higher in women expressing milk with a breast pump. In addition, *Pseudomonas* was only detected in samples from women using breast pumps [28]. Therefore, great emphasis must be placed on the appropriate instruction of future donors to ensure the microbiological safety of HM donated to the bank. However, not all bacteria can enter milk from the external environment. Anaerobic strains, in particular, can enter the milk, e.g., through the enterogastric pathway, with the help of dendritic cells, macrophages, or lymphocytes [29].

To summarise, in the microbiological analyses of all samples (n = 139), *S. epidermidis* (68.35%)*, S. mitis/oralis* (10.79%), and *S. aureus* (6.47%) were detected in the largest number of samples at the Regional Human Milk Bank in Warsaw. They are typical bacteria of the oral cavity and skin. Despite the differences in the results between individual research groups, the same relationship was also noticed in other medical institutions and the HMB. Serafini et al., in their studies, showed that *S. epidermidis* was detected in 20.59% of samples, which was also one of the most frequently detected strains in the Brazilian milk bank, and *S. aureus* in 7.35% of unpasteurised milk [30]. However, Taiwanese researchers found the presence of coagulase-negative *Staphylococcus* in 64.3% of samples, and *S. aureus* in 2.2% [19]. Ifeanyi OC Obiajuru et al. [31] found *S*. *epidermidis* in 56.7% and *S. aureus* in 9.3% of samples, which is closer to our results. Moreover, infection with *S. aureus* was observed more often in women with worse health during pumping [19,31]. In the Norwegian Milk Bank, *S. epidermidis* was found most often (85%), and *S. aureus* was found in 13.1% of samples [20]. Contamination with these strains deserves much attention, because *S. epidermidis/aureus* has the ability to form a biofilm on biotic and abiotic surfaces [32]. The bottle in which milk is kept before and after pasteurization can be a good surface for biofilm forming.

Contamination with Gram-negative rods was observed much less frequently than positive and negative-coagulase staphylococci. They were found in no more than 3% of our samples. The results of donors in the world’s Human Milk Banks and medical institutions are very diverse in this group of bacteria. In a Human Milk Bank in Taiwan, contamination with Gram-negative rods (especially *E. coli*) was found in 22.9% of samples, and in Nigeria in 50%, which is a much higher value than in the RHMB in Warsaw. Similar levels of coliform bacteria were recorded in the Milk Bank in Oslo: *E. coli* 0.45%, *Klebsiella* 2%, and *Enterobacter* 1.5%. Serra et al. obtained similar results (4.3% *E. coli*). The remaining bacteria were also at a similar level of detection [9,19,20,31].

Contamination of *Bacillus cereus* also deserves attention. It is a bacterium that occurs in a vegetative form and as heat-resistant spores that are able to survive the pasteurization process, e.g., the holder method. In French HMBs, *Bacillus cereus* was prominent in milk from donors and was detected both before and after pasteurization [33,34]. A study on donor women in Taiwan also indicated that samples that were pasteurized showed an increase in *Bacillus* sp. [19]. Jandova et al. [35] show that *B. cereus* is a major cause of HMB discard, as they were found in most samples after pasteurization. Although the causality has not been proven, and simulation studies have shown a low risk of infection with *Bacillus* sp., the milk of donors is indicated as the main source of possible infection, which can even lead to sepsis and death [36]. French scientists suggested the use of more sensitive methods of detecting this type of bacteria in breast milk, especially in new donors, which could lead to lower milk losses due to the early elimination of contaminated samples. However, modern techniques are very costly [37]. In our study, we found the presence of *Bacillus* spp. in 4.32% of the samples. Due to the ability of the bacilli to spore, it may not be possible to detect this bacterium by standard inoculation methods, and therefore the results could be false negatives. In addition, the possibility of transformation from a spore to a vegetative form of the stored sample after pasteurization should be taken into account. The Human Milk Bank Foundation recommends that in every case when a woman’s bacteria titer is close to 10^5^ CFU/mL, selection tests, especially for spore-forming bacteria or some toxin-producing strains, should be performed, which is an additional safeguard against allowing milk contaminated with *Bacillus* spp. into the pool intended for pasteurization [14]. In addition, our previous research shows that high pressure can be a good alternative to holder pasteurization, because we did not observe any growth of *Bacillus cereus* after pressurisation [38].

As is known, *Lactobacillus* are one of the most common strains in human milk, but our results show that only 0.72% of women had probiotic bacteria detected. Culture-independent techniques used by other research groups (quantitative polymerase chain reaction (qPCR) and, later on, next-generation sequencing (NGS), mostly based on 16S rRNA gene) have shown that human milk contains a much greater variety of bacteria than was originally thought. The use of such techniques confirmed the dominance of staphylococci and streptococci as well as the presence of Lactic acid bacteria, propionibacteria, and bifidobacterial, which had not been previously cultivated by classic methods, e.g., especially anaerobic species [5,6,11,29,39]. Therefore, the results of our research may be to some extent underestimated, both quantitatively and qualitatively, due to the fact that it is difficult to culture, for example, anaerobic bacteria. These values are in fact probably much higher; however, due to technical limitations of inoculation methods at the first step of analysis of our samples, these strains were not grown in donor samples. If we inoculate samples, for example, on selective medium for lactobacilli, the MALDI Biotyper Bruker (Bruker Daltonik GmbH, Germany) will recognise those probiotic bacteria in more women.

Human milk ensures proper development, especially for low-birth-weight premature babies, who are more at risk of infection than full-term babies. When the infant’s mother is unable to feed her child for various reasons, donating human milk is a much better solution than using formulas based on cow’s milk. Although the milk subjected to holder’s pasteurization contains fewer nutrients and biological components than raw milk, and the fact that the low temperature eliminates not only potentially pathogenic microorganisms but also pro-health microorganisms, it is the most beneficial food possible [40,41]. It is not fully established how donor human milk modulates the microbiome of a premature infant, but some authors suggest that the presence of the dead, damaged bacterial cells in milk may be considered as the so-called “Para-probiotics” or “ghost probiotics” and can induce a response from the immune system [29]. On the other hand, Fernandez et al. [42] proposed that the fresh milk could be thoroughly analysed for CMV and served raw. In addition, he suggested that pasteurized milk should be supplemented with probiotic bacteria, which could increase the ability to modulate the newborn’s microbiome; each mother has a unique microbiota, and inoculation of maternal microorganisms in the donor’s milk could provide these microorganisms to the mothers’ infants [42,43].

## 5. Conclusions

Guidelines for microbiological purity of donated milk vary greatly between banks in different European countries, and detailed microbiological testing depends on the resources of individual hospital laboratories. Microbiological analysis applied in Regional Human Milk Bank in Warsaw allowed for quick and precise screening of characteristics of human milk microbiota. Implemented procedures have also detected pathogenic microorganisms, and the corrective actions resulted in elimination of the risk.

This was crucial for further development of human milk banking in Poland and clinical significance of donor milk for the newborn. In addition, with the growing knowledge of the benefits of raw human milk to premature infants, more efficient and detailed quality control of donated milk may lead to a change in approach and distribution of unpasteurized donor milk by milk banks. However, such an approach would require the establishment of standardized and stringent requirements for expressing milk, which must be met in order to receive donor milk as safely as possible, preserving the benefits of human milk microbiota.

## Figures and Tables

**Table 1:** Amounts of milk collected in Regional Human Milk Bank (RHMB) in Warsaw per year.

Year	Total Collected Amount, L	Total Distributed Amount, L	Total Disposed Amount, L
**2016**	308.14	287.86	8.60
**2017**	363.00	313.92	5.00
**2018**	301.95	331.25	8.41
**2019**	472.50	463.45	9.10
**Total**	1445.59	1396.48	31.11

**Table 2:** Number of new donors’ milk samples per year and distribution of the results of microbiological analysis of the raw milk samples.

Year	Number of New Donors	Frequency of Bacterial Contamination of Sample of Donor Milk							
		First Analysis				Repeated Analysis			
		Number of Analysed Samples	Negative	Positive	Rejected	Number of Analysed Samples	Negative	Positive	Rejected
**2016**	25	25	3 (12.00%)	22 (88.00%)	3 (12.00%)	14	2 (14.29%)	12 (85.71%)	4 (28.57%)
**2017**	20	20	5 (25.00%)	15 (75.00%)	3 (15.00%)	14	6 (42.86%)	8 (57.14%)	3 (21.43%)
**2018**	26	26	3 (11.54%)	23 (88.46%)	2 (7.69%)	4	1 (25.00%)	3 (75.00%)	0 (0.00%)
**2019**	25	25	7 (28.00%)	18 (72.00%)	2 (8.00%)	11	4 (36.36%)	7 (63.64%)	0 (0.00%)
**Total**	96	96	18 (18.75%)	78 (81.25%)	10 (10.42%)	43	13 (30.23%)	30 (69.77%)	7 (16.28%)

Negative—sample of microbiologically clean milk; Positive—sample with one or more species of commensal and/or pathogenic bacteria; Rejected—sample with the level of bacteria above the RHMB standard.

**Table 3:** Microbiological findings in 139 breast milk samples from donors.

Microorganism	First Analysis				Repeated Analysis				Total			
	Positive		Above the Standard *		Positive		Above the Standard *		Positive		Above the Standard *	
	N	%	N	%	N	%	N	%	N	%	N	%
*Staphylococcus epidermidis*	70	72.92%	4	4.17%	25	58.14%	0	0.00%	95	68.35%	4	2.88%
*Staphylococcus aureus*	7	7.29%	4	4.17%	2	4.65%	1	2.33%	9	6.47%	5	3.60%
*Staphylococcus warneri*	2	2.08%	0	0.00%	3	6.98%	0	0.00%	5	3.60%	0	0.00%
*Staphylococcus haemolyticus*	4	4.17%	1	1.04%	2	4.65%	0	0.00%	6	4.32%	1	0.72%
*Staphylococcus hominis*	2	2.08%	0	0.00%	0	0.00%	0	0.00%	2	1.44%	0	0.00%
*Staphylococcus pasteuri*	0	0.00%	0	0.00%	1	2.33%	0	0.00%	1	0.72%	0	0.00%
*Staphylococcus capitis*	1	1.04%	0	0.00%	0	0.00%	0	0.00%	1	0.72%	0	0.00%
*Staphylococcus lugdunensis*	3	3.13%	0	0.00%	0	0.00%	0	0.00%	3	2.16%	0	0.00%
*Streptococcus mitis/oralis*	12	12.50%	0	0.00%	3	6.98%	0	0.00%	15	10.79%	0	0.00%
*Streptococcus salivarius*	5	5.21%	0	0.00%	1	2.33%	0	0.00%	6	4.32%	0	0.00%
*Streptococcus pluranimalium*	1	1.04%	0	0.00%	0	0.00%	0	0.00%	1	0.72%	0	0.00%
*Streptococcus urinalis*	1	1.04%	0	0.00%	1	2.33%	0	0.00%	2	1.44%	0	0.00%
*Streptococcus peroris*	1	1.04%	0	0.00%	0	0.00%	0	0.00%	1	0.72%	0	0.00%
*Streptococcus vestibularis*	3	3.13%	0	0.00%	0	0.00%	0	0.00%	3	2.16%	0	0.00%
*Streptococcus parasanguinis*	0	0.00%	0	0.00%	1	2.33%	0	0.00%	1	0.72%	0	0.00%
*Klebsiella oxytoca*	0	0.00%	0	0.00%	2	4.65%	2	4.65%	2	1.44%	2	1.44%
*Escherichia coli*	1	1.04%	1	1.04%	0	0.00%	0	0.00%	1	0.72%	1	0.72%
*Enterococcus faecalis*	5	5.21%	1	1.04%	1	2.33%	0	0.00%	6	4.32%	1	0.72%
*Bacillus* spp.	2	2.08%	2	2.08%	4	9.30%	4	9.30%	6	4.32%	6	4,32%
*Lactobacillus gasseri*	1	1.04%	0	0.00%	0	0.00%	0	0.00%	1	0.72%	0	0.00%
*Chryseobacterium indologenes*	0	0.00%	0	0.00%	1	2.33%	0	0.00%	1	0.72%	0	0.00%
*Acinetobacter baumannii*	1	1.04%	1	1.04%	0	0.00%	0	0.00%	1	0.72%	1	0.72%
*Corynebacterium simulans*	1	1.04%	0	0.00%	0	0.00%	0	0.00%	1	0.72%	0	0.00%
*Candida albicans*	1	1.04%	0	0.00%	0	0.00%	0	0.00%	1	0.72%	0	0.00%
Total analysed women	96				31				96			
Total analysed samples	96				43				139			

In some samples, two or more bacteria were detected. * Above the standard = according to Human Milk Bank Foundation recommendation: the total number of bacteria in the sample <10^5^ CFU/mL; the number of coagulase-positive staphylococci <10^4^ CFU/mL; the number of coliform bacteria <10^3^ CFU/mL.

## Data Availability

Not applicable.
